# Auditory verbal hallucinations in schizophrenia and post-traumatic stress disorder: common phenomenology, common cause, common interventions?

**DOI:** 10.3389/fpsyg.2015.01071

**Published:** 2015-07-28

**Authors:** Simon McCarthy-Jones, Eleanor Longden

**Affiliations:** ^1^Department of Psychiatry, Trinity College DublinDublin, Ireland; ^2^Institute of Psychology, Health and Society, University of LiverpoolLiverpool, UK

**Keywords:** abuse, dissociation, hallucinations, hearing voices, memory, PTSD, psychosis, traumatology

## Abstract

Auditory verbal hallucinations (AVH: ‘hearing voices’) are found in both schizophrenia and post-traumatic stress disorder (PTSD). In this paper we first demonstrate that AVH in these two diagnoses share a qualitatively similar phenomenology. We then show that the presence of AVH in schizophrenia is often associated with earlier exposure to traumatic/emotionally overwhelming events, as it is by definition in PTSD. We next argue that the content of AVH relates to earlier traumatic events in a similar way in both PTSD and schizophrenia, most commonly having direct or indirect thematic links to emotionally overwhelming events, rather than being direct re-experiencing. We then propose, following cognitive models of PTSD, that the reconstructive nature of memory may be able to account for the nature of these associations between trauma and AVH content, as may threat-hypervigilance and the individual’s personal goals. We conclude that a notable subset of people diagnosed with schizophrenia with AVH are having phenomenologically and aetiologically identical experiences to PTSD patients who hear voices. As such we propose that the iron curtain between AVH in PTSD (often termed ‘dissociative AVH’) and AVH in schizophrenia (so-called ‘psychotic AVH’) needs to be torn down, as these are often the same experience. One implication of this is that these trauma-related AVH require a common trans-diagnostic treatment strategy. Whilst antipsychotics are already increasingly being used to treat AVH in PTSD, we argue for the centrality of trauma-based interventions for trauma-based AVH in both PTSD and in people diagnosed with schizophrenia.

## Introduction

Auditory verbal hallucinations (AVH), hearing voices in the absence of appropriate external stimuli, are reported by approximately three in four people diagnosed with schizophrenia (Thomas et al., 2007). They are also found with notable regularity in post-traumatic stress disorder (PTSD; Butler et al., 1996; Anketell et al., 2010; Brewin and Patel, 2010). In the most studied sub-group of people with PTSD, military veterans, recent studies have reported an AVH prevalence ranging from 50–67% (Holmes and Tinnin, 1995; Anketell et al., 2010; Brewin and Patel, 2010).

In this paper we will first argue that AVH in PSTD (PTSD-AVH) have a qualitatively similar phenomenology to AVH in schizophrenia (SZ-AVH). We will then demonstrate that whilst traumatic life events are associated with AVH in PTSD by definition, they are also associated with AVH in schizophrenia. Next, we will show that the phenomenology of AVH in both schizophrenia and PTSD relate to earlier traumas, when present, in a comparable fashion, and examine how this can be accounted for. Finally, we will explore the implications of this for the treatment of SZ-AVH and PTSD-AVH.

## The Phenomenology of AVH in PTSD and Schizophrenia

### Arguments for Dissimilarities

Arguments that the phenomenology of PTSD-AVH is qualitatively distinct from that of SZ-AVH are not supported by contemporary research. One proposition is that PTSD-AVH are ego-syntonic ‘pseudo hallucinations’ (recognized as one’s own cognitions), whereas SZ-AVH are ego-dystonic ‘true hallucinations’ (experienced as externally generated). Based on their finding that all thirty PTSD-AVH patients they interviewed regarded their AVH as a manifestation of their own thoughts, Brewin and Patel (2010, p. 424) argued that PTSD-AVH are a dissociative phenomenon, distinct from the “psychotic processes” that result in SZ-AVH. However, whilst Anketell et al. (2011, p. 93) found that some patients with PTSD did indeed recognize some congruence between the content of their AVH and their own thoughts, the majority reported AVH that were “experienced as ego-dystonic or alien to the extent that they were endowed with the qualities of an entirely separate entity or person.” Just as some PTSD-AVH may be ego-dystonic, some SZ-AVH can be ego-syntonic, with McCarthy-Jones et al. (2014) finding that 34% of people diagnosed with schizophrenia-spectrum disorders with AVH said some of their AVH could have been their own voice/thoughts. Instead of being diagnostically specific, the extent to which voices are ego-syntonic or ego-dystonic appears comparably heterogeneous in PTSD-AVH and SZ-AVH.

As is well known, Schneider (1959) argued that certain types of voices (running commentaries and voices conversing) were diagnostic of schizophrenia, a distinction that was still reflected in DSM-IV-Tr (American Psychiatric Association, 2000). However, there are no differences between rates of running commentaries or voices conversing between patients with PTSD and patients with psychotic disorders (Scott et al., 2007), and Schneider’s position has been abandoned in DSM-5 (American Psychiatric Association, 2013).

Another argument is that PTSD-AVH typically take the form of auditory flashbacks (re-experiencing of auditions surrounding the trauma), whereas SZ-AVH do not. Such a proposition is contradicted by the finding that PTSD-AVH are phenomenologically distinct from auditory flashbacks where the person acts or feels as if the traumatic event is actually recurring (Butler et al., 1996). Furthermore, PTSD-AVH are not confined to episodes of re-experiencing, but remain present continuously as in SZ-AVH (Braakman et al., 2009).

A final argument proposes that the content of AVH in patients with a psychosis subtype of PTSD is almost always trauma-related, whereas in schizophrenia they are not (Scott et al., 2007; Braakman et al., 2009). However, this argument is not empirically supported (Hardy et al., 2005; Anketell et al., 2010; Corstens and Longden, 2013), as we address in Section “Trauma, SZ-AVH, and PTSD-AVH.”

### Direct Phenomenological Comparisons

Studies directly comparing the phenomenology of PTSD-AVH and SZ-AVH have found no notable differences. To our knowledge, there have only been two such studies (Scott et al., 2007; Jessop et al., 2008). The first of these compared the AVH of 17 PTSD patients with those of 16 patients with psychotic disorders, and found no differences between the groups in the rates of Schneiderian-type voices (Scott et al., 2007). The study did find that command AVH were more common in SZ-AVH (63%) than PTSD-AVH (37%), and that only hearing internally located voices was more common in PTSD-AVH (35%) than SZ-AVH (11%).

The second study of this question, although drastically underpowered (*N* = 18; schizophrenia *n* = 5, PTSD *n* = 13) and hence largely reliant on indicative descriptions, found that the majority of both groups heard both externally and internally located AVH (Jessop et al., 2008). Around half of each group reported perceptually vivid voices. In terms of differences, Jessop et al. (2008) found that the frequency and duration of AVH were lower in PTSD than in schizophrenia, and that participants with PTSD reported greater subjective distress than those with schizophrenia. The study also found that command hallucinations and derogatory themes were more common in PTSD, contradicting the findings of Scott et al. (2007) There is hence no clear evidence for qualitative differences between PTSD-AVH and SZ-AVH from extant (albeit underpowered) research.

### Indirect Phenomenological Comparisons

Due to the small numbers of patients involved in direct comparison studies, PTSD-AVH may be compared with SZ-AVH based on the wider phenomenological literature in this area. To do this, we characterized AVH in schizophrenia using the three largest studies of SZ-AVH (Nayani and David, 1996, *N* = 100; Corstens and Longden, 2013, *N* = 100; McCarthy-Jones et al., 2014, *N* = 199). Studies of the phenomenology of AVH in PTSD have been notably smaller. To characterize the phenomenology of PTSD-AVH we drew on the three largest detailed studies of this phenomenon we were aware of (Scott et al., 2007, *N* = 20; Anketell et al., 2010, *N* = 20; Brewin and Patel, 2010, *N* = 30). In one instance where a notable aspect of the phenomenology of AVH was described in the PTSD-AVH studies but not SZ-AVH (and vice versa for another aspect of AVH phenomenology), we drew on smaller studies to gather information about this property of AVH (Kent and Wahass, 1996; Hamner et al., 2000). The resultant comparisons are shown in **Table 1**. As can be seen PTSD-AVH and SZ-AVH appear highly similar phenomenologically, and where differences do appear, these are quantitative rather than qualitative.

**Table 1:** A phenomenological comparison of auditory verbal hallucinations (AVH) between post-traumatic stress disorder (PTSD) and schizophrenia.

Property	PTSD	Schizophrenia-spectrum disorders
Unpleasant voices	Present, more common than pleasant voices (Anketell et al., 2010; Brewin and Patel, 2010)	Present, more common than pleasant voices (Nayani and David, 1996; Corstens and Longden, 2013; McCarthy-Jones et al., 2014)
Pleasant voices	Present, but less common than negative voices (Anketell et al., 2010; Brewin and Patel, 2010)	Present, but less common than negative voices (Nayani and David, 1996; Corstens and Longden, 2013; McCarthy-Jones et al., 2014)
Voices issue commands?	Yes (Scott et al., 2007; Anketell et al., 2010)	Yes (Nayani and David, 1996; Corstens and Longden, 2013; McCarthy-Jones et al., 2014)
Commands to hurt self	Yes (Anketell et al., 2010)	Yes (Kent and Wahass, 1996)
Link to trauma	Only 40% linked voices to earlier traumas (Anketell et al., 2010)	Some but not all linked to trauma (Hardy et al., 2005; Corstens and Longden, 2013)
Location	Mostly internally located, but some external and some in both locations (Scott et al., 2007; Anketell et al., 2010)	Approximately equal mix between internal, external, and both (McCarthy-Jones et al., 2014)
Clarity	As clear as a real voice (Anketell et al., 2010)	Typically clear or very clear (McCarthy-Jones et al., 2014)
Unclear voices	In addition to clear content, may include “garbled voices” (Hamner et al., 2000)	In addition to clear content, may include “nonsense voices” (McCarthy-Jones et al., 2014)
Experienced as manifestation of own thoughts	Present in some (Brewin and Patel, 2010)	Present in some (McCarthy-Jones et al., 2014)
Gender	Voices are mainly male (Brewin and Patel, 2010)	Voices are mainly male (Corstens and Longden, 2013; McCarthy-Jones et al., 2014)
Identity of voice	Most recognized as being that of person known in the real world to the hearer (Anketell et al., 2010; Brewin and Patel, 2010)	Approximately equal mix between known, unknown, and both (McCarthy-Jones et al., 2014)
Form of address	Most commonly use ‘you’ (Brewin and Patel, 2010)	Most commonly use ‘you’ (Corstens and Longden, 2013; McCarthy-Jones et al., 2014)
Frequency	Voices heard many times a day (Brewin and Patel, 2010)	Most likely to be constantly with the person (McCarthy-Jones et al., 2014)
Control	Around a third can control their voices (Brewin and Patel, 2010)	Fifty-one percent can exercise some control over their voices (Nayani and David, 1996)
Number of voices heard	May hear one or more voices, and groups of voices (Anketell et al., 2010; Brewin and Patel, 2010)	May hear one or more voices, and groups of voices (Nayani and David, 1996; Corstens and Longden, 2013; McCarthy-Jones et al., 2014)

### Summary

Available studies support the argument that PTSD-AVH and SZ-AVH are qualitatively similar in phenomenological terms. This implies that a description of an AVH in isolation should not permit a reliable discernment as to whether it was reported by someone diagnosed with either PTSD or schizophrenia. This appears to be the case. For example, take the following description of an AVH:

One [voice] tells me to hurt myself… And I did in the past… It’s very nasty…always criticizing…continually at you…‘try and do yourself in,’ ‘commit suicide’…they try and tell you how to do it… there’s never anything good…Through time a girl appeared… She was more peaceful and more subdued… saying to me… ‘just don’t pay attention to him’… It’s controlling my life… sometimes it’s a battle with them… just to survive.

We submit that only the referenced source of this quote (Anketell et al., 2011, p. 92) would allow the reader to correctly infer whether this was a voice heard by a person diagnosed with PTSD or schizophrenia. Although we cannot rule out that a large-scale study explicitly comparing AVH between people diagnosed with PTSD and people diagnosed with schizophrenia may highlight some important differences, it appears that PSTD-AVH and SZ-AVH frequently share a common phenomenology.

## Trauma, SZ-AVH, and PTSD-AVH

### Is Trauma Associated with SZ-AVH and PTSD-AVH?

Whilst PTSD-AVH have their roots in traumatic experiences by definition, the role of trauma in SZ-AVH has historically been drastically underestimated (Read et al., 2005). There is now good evidence for an association between traumas (particularly, but not exclusively, childhood abuse) and SZ-AVH (Read et al., 2003; McCarthy-Jones, 2011; Sheffield et al., 2013), as well as between trauma and AVH in the general population (Bentall et al., 2012). Dose–response relations between trauma and AVH (Bentall et al., 2012), and between trauma and psychosis in general (Varese et al., 2012b), suggest a causal relation. Commonalities in the neurological effects of trauma and the neurological changes associated with AVH are also consistent with a causal relation (Read et al., 2014). It is hence plausible that PTSD-AVH and SZ-AVH share a common phenomenology because they often share a common cause.

### Are PTSD-AVH and SZ-AVH Phenomenologically Related to Trauma in Similar Ways?

Studies of people diagnosed with PTSD, as well as of people diagnosed with schizophrenia, have examined if and how the content of AVH may relate to previous traumatic events in the person’s life, if present. Such studies have typically lacked the methodological rigor to allow concerns over the reliability and validity of links reported to be satisfactorily addressed.

The methodologically strongest of these studies, by Hardy et al. (2005), examined the relation between trauma and the content of hallucinations in patients with non-affective psychosis. Participants were assessed three months apart, being asked at baseline about the phenomenology of their hallucinations (which were predominantly, but not exclusively, AVH), and then asked at 3-month follow up about childhood traumatic experiences (using the Trauma History Questionnaire, Green, 1996). A bespoke coding framework was devised to classify the content of traumas and hallucinations, with each coding being independently made by two separate raters to allow assessment of inter-rater reliability. First, content ratings were made based on whether there was a literal correspondence between the content of trauma and the content of hallucinations. For example, a content association was deemed present if a person who was threatened with a gun experienced visions of guns. There was 100% inter-rater agreement on content associations. Second, both hallucinations and traumas were rated on four themes (humiliation, intrusiveness, threat, and guilt) to assess indirect associations between them. Clear operational definitions were created for these themes. The presence of these themes in hallucinations and traumas were assessed separately meaning that raters were blind to any associations of traumas with hallucinations. Kappa statistics for inter-rater reliabilities for the assessment of these themes in traumatic life events ranged from 0.45 to 0.62, and from 0.45 to 0.64 for these themes in hallucinations. These hence represented only moderate agreement, raising some concerns over the reliability of these judgements.

Of the 75 participants in the study, 40 reported having experienced trauma (e.g., sexual abuse, bullying, military combat). Of these 40 patients, 5 (12.5%) were found to have direct content associations between their trauma and their hallucination, 23 (57.5%) were found to have indirect, thematic associations between their trauma and their hallucinations (this figure included the five participants with direct content associations, as they also had thematic correspondences between their traumas and hallucinations), and 17 (42.5%) had no apparent relation between the content of their hallucinations and their trauma. This suggested that a subset of people diagnosed with a psychotic disorder have experienced trauma, and that a subset of these have links between their trauma and the content of their hallucinations. In this study, this subset of a subset who had links between their trauma and the content of their hallucinations represented 30.6% of the overall sample of patients with non-affective psychoses.

It is possible that this study underestimated links between AVH-content and traumatic life events by only inquiring about life-events deemed traumatic by Green’s (1996) Trauma History Questionnaire. A more recent study by Corstens and Longden (2013) looked not just at trauma in relation to AVH-content, but at a wider range of emotionally overwhelming events covering a range of social and emotional problems. This was done with 100 people who heard voices (80% of who had received a diagnosis of schizophrenia or other psychotic diagnosis). Themes of AVH-content were coded using a variety of coding schemes derived from clinical expertise, existing theoretical and empirical literature, and the work of Romme and Escher (2000). For example, codes applied to the key themes of voice-hearing were problems and conflicts relating to: shame and guilt, sexual identity, self-esteem, anger, and attachment and intimacy. One author initially coded all interviews, and to confirm agreement and consistency in this coding system, a subset of 30 constructs were selected at random and rated on the same criteria by both the second author and an independent rater external to the study. However, formal kappa ratings were not reported, and the reliability and validity of the ensuing conclusions are hard to assess. The study found that the vast majority of the sample (94%) had AVH whose content could be related to earlier emotionally overwhelming events, with both voices and adverse events often having emotional congruence (e.g., both involving low self-worth, anger, shame, and guilt) and identity congruence (e.g., both involving a family member, a past abuser).

A range of other studies have also produced findings consistent with the hypothesis that a significant proportion of patients diagnosed with schizophrenia-spectrum diagnoses have AVH with content that relates to earlier adversities. Reiff et al. (2012) interviewed 21 people who had both been diagnosed with schizophrenia-spectrum diagnoses (including hallucinations/delusions) and had experienced physical or sexual abuse as a child. They found that 14 (67%) patients had links between their child abuse and the content of their hallucinations/delusions. For example, one participant who had suffered child sexual abuse by their step-father later heard the hallucinated voice of the step-father; another who was raped in childhood later heard a hallucinated voice that threatened rape. Thompson et al. (2010) found that, in a population at ultra-high risk for psychosis, having a history of sexual trauma increased the odds ninefold that sub-threshold psychotic experiences with sexual content would be experienced. Raune et al. (2006) found attributes of stressful events in the year preceding psychosis onset were associated with core themes of both delusions and hallucinations, and Smith et al. (2006) found individuals with greater levels of depression and lower self-esteem had AVH of more intensely negative content.

All this suggests that a notable subset of people diagnosed with schizophrenia-spectrum disorders that who experience AVH have voices whose content is related to earlier traumatic or adverse life-experiences. Hardy et al.’s (2005) study suggests such relations may exist in around a third of such patients, with Corstens and Longden’s (2013) work suggesting that if the concept of trauma is expanded to cover a wider range of adverse or emotionally overwhelming events, that this percentage may be much higher. More detailed research is needed to create a more precise estimate. Nevertheless, what emerges clearly is that the nature of the relation between trauma and the content of AVH in psychosis is not typically one of re-experiencing (Fowler et al., 2006) but instead involves a direct or indirect, thematic relation. This is not to say that some SZ-AVH are not forms of re-experiencing, as McCarthy-Jones et al. (2014) found that 39% of patients with schizophrenia-spectrum diagnoses reported that their voices were in some way “replays” of memories of previous conversations they’d had/heard (although it was not enquired if these related to trauma), and that of these patients, 23% (i.e., 9% of the overall sample) said that these replays were identical to these earlier conversations (although such voices only completely made up 14% of these people’ voices). Forms of direct re-experiencing may hence make up some SZ-AVH, but this appears to be a small corner of the experience.

A similar pattern of findings – predominantly indirect/thematic relations between trauma and AVH content – emerges from the PTSD research literature. Anketell et al. (2010) found 40% of PTSD patients directly related their voices to past experiences of abuse or trauma. For example, one patient who had been physically assaulted by two men subsequently heard the voices of two men saying, “We’ll kill you.” For others, the link between the content of the trauma and the AVH was not immediately evident, with one man who had suffered physical and sexual abuse reporting three distinct voices all “making a case for self-harm and suicide” that appeared to reflect the internalized message given to him by the abuser. This echoes the indirect, thematic link found between many SZ-AVH and trauma by Hardy et al. (2005). However, methodological safeguards to validity and reliability, as employed by Hardy et al. (2005) were not employed in this study.

Consistent with this finding though, other research has also found that PTSD-AVH appear to often have themes which indirectly reflect the nature of the trauma (e.g., derogatory comments referring to the participant’s perceived inadequacy). For example, Jessop et al. (2008) found 46% of patients with PTSD reported an association between their AVH and earlier traumatic experiences, but that the content was not typically a direct reflection of a traumatic event. Instead, the AVH included themes that were understandable in the context of trauma (e.g., “you are hopeless,” “you deserve to die,” in the case of childhood physical and sexual abuse) or related to derogatory comments previously said to them. Similarly, Butler et al. (1996) report that AVH in persons with PTSD are often congruent with the trauma, but not identical to it (e.g., a dead soldier calling to the patient to kill himself). This moves the concept of AVH in PTSD away from PTSD-AVH as simple re-experiencing.

To illustrate how voices may be related to traumas in PTSD, but are clearly not re-experiencing of what actually happened consider the example of David (not his real name; taken from Bleich and Moskowits, 2000). David was a 25-year old tank crew member in the Lebanon War, when an artillery shell hit his tank. He was picked up and thrown by the blast. He survived but other crew members were killed. In the days immediately afterward he became restless and anxious. Worrying thoughts about the event kept coming into his mind, he became agoraphobic, emotionally volatile, suffered sleep disruption, and he avoided weapons whenever he could. Several weeks later he started hearing voices. They were the voices of his dead comrades, accusing David of betraying them by leaving them and remaining alive. They commanded him to join them by committing suicide. These were obviously words never actually spoken to David by his comrades whilst they were alive (i.e., they were not direct re-experiencing of actual speech), but the content of these voices was clearly related to his trauma. The content of AVH in PTSD hence seems to have a similar relationship to trauma as in SZ-AVH; related but different.

Only one study has directly compared the relationship of AVH-content to trauma in PTSD and schizophrenia. Scott et al. (2007) found that five out of 20 patients with PTSD reported that the content of their hallucinations represented a past psychologically traumatic experience (e.g., hearing the voice of a perpetrator), the trauma being sexual abuse in all cases. However, Scott et al. (2007, p. 46) they found that “Hallucinations representing psychological trauma did not occur in patients with psychotic disorder.” No details were given on the methodology used to assess links between trauma and hallucination content. Given that studies employing more detailed, rigorous, and systematic approaches to assessing links between trauma and AVH-content in patients with schizophrenia-spectrum diagnosis have found links in many cases (see above; Hardy et al., 2005; Reiff et al., 2012; Corstens and Longden, 2013), it appears Scott et al.’s (2007) failure to find associations may have been due to methodological limitations.

In the context of methodological problems of extant studies, it may nevertheless be tentatively concluded that AVH with a basis in trauma, whether they occur in the context of a PTSD diagnosis or a schizophrenia diagnosis, often (but not always) relate to this trauma, and typically do so in a way that is indirect/thematic rather than taking the form of re-experiencing. However, there is a need for more methodologically rigorous studies to examine the nature and prevalence of relations between the content of AVH and earlier traumas, when present.

## How Can We Account for the Varied Ways AVH are Related to Trauma?

A range of mechanisms have been proposed to account for the relation between traumatic life events and the later emergence of AVH, which span a range of levels of explanation (for reviews see, McCarthy-Jones, 2012; Read et al., 2014). These include the biological (altered hypothalamic–adrenal–pituitary axis activity, altered dopaminergic transmission), cognitive (source monitoring errors, content memory deficits), and affective (emotional suppression, altered emotion recognition). Here, we will first examine how a well-established model of re-experiencing in PTSD may be used to account for AVH (in both PTSD and schizophrenia) that are linked to earlier traumatic events but which are not direct re-experiences, before briefly considering how other relations may be possible.

### AVH as a Re-Experiencing Phenomenon

Ehlers and Clark (2000) argue that re-experiencing in PTSD results from intrusions from memory, due to traumatic experiences not being laid down in autobiographical memory in a consolidated fashion. Specifically, they propose that during trauma exposure cognitive processing becomes data-driven rather than conceptually driven. This altered form of processing has been referred to as dissociative (meaning the stimuli is processed in an unintegrated manner), and has been proposed to result in what Brewin (2001) has termed a situationally accessible memory (SAM), which can be difficult to recall voluntarily, but which can more easily be involuntarily triggered into consciousness when stimuli associated with the original trauma are encountered.

Modeling SZ-AVH as a form of re-experiencing symptom would be consistent with much SZ-AVH research. The proposal that re-experiencing results from the context of the traumatic event not being encoded (Ehlers and Clark, 2000), fits with evidence of context memory deficits in SZ-AVH (Waters et al., 2006). The dose–response relationship between trauma and AVH (Bentall et al., 2012) is congruent with Ehlers and Clark’s (2000) proposal that a second traumatic event is even less likely to be integrated into autobiographical memory due to it triggering of memories of the original exposure and impairing the person’s ability to conceptually process the event. Re-experiencing symptoms taking the form of intrusions from memory also have a strong sensory component, consistent with the phenomenology of many (although not all) AVH in both PTSD and schizophrenia. Yet, the question remains as to why AVH (in both PTSD and in SZ) are not typically re-experiences of trauma, but do have indirect or direct links to trauma.

### Existing Attempts to Account for the Relation between AVH and Traumatic Events

We are only aware of one previous attempt to explain why utterances heard during a traumatic event may inform the content of AVH, but not be such words verbatim. Brewin et al. (1996) discuss how SAMs may be altered. SAMs cannot be deliberately accessed, occur automatically when the person is in a context where physical cues or meanings are encountered similar to those of the earlier traumatic situation, and are associated with a flashback type re-experiencing. Brewin et al. (1996, p. 678) argue that SAMs can be “automatically altered or added to whenever some or all of the information they contain happens to be paired with changes in concurrent bodily states or contents of consciousness.” They further claim that (beneficial) changes to the SAM would be expected to follow changes such as the absolution of responsibility for the trauma, whereas negative changes would be expected if subsequent information changes the perception of the event in a negative way (further self-shaming information). They suggest that this may occur through an associative process, e.g., a SAM of the event occurs and is followed by the thought, “I should kill myself.” This new thought then becomes a SAM in itself, and forms the basis of an AVH. This implies, although Brewin et al. (1996) do not explicitly make this point, that verbally accessible memories (VAMs) of the trauma (a series of autobiographical memories which can be deliberated and progressively edited) may interact and alter the SAMs of the event in a dynamic way.

### Applying Theories of Reconsolidated Memory to AVH

Contemporary models of memory may also be employed to explain why the content of AVH is related to an earlier event, but not identical to it. Consolidation theory assumes that memories are labile for a limited time after encoding, but then consolidation occurs and these memories become fixed, or at least resistant to change (McGaugh, 2000). However, it has long been known that episodic memories can be reconstructed (Bartlett, 1932), and altered by post-event information. For instance, providing participants with misleading information after the encoding of an event can revise the original memory (Loftus, 2005). Yet, it was only more recently that reactivation of the memory was considered as a potential prerequisite for memory change. Hupbach et al. (2007) showed that modification of consolidated episodic memories is critically dependent upon their reactivation. It hence appears that after reactivation, apparently stable memories may re-enter an unstable state in which they are modifiable, requiring another phase of stabilization, (‘reconsolidation’), and that in this transient plastic state, memories are susceptible to the incorporation of new information (Wichert et al., 2013).

Consistent with this, memories are now conceptualized as “transitory dynamic mental constructions” (Conway and Pleydell-Pearce, 2000, p. 261) that are “created in the present moment, according to the demands of the present” (Fernyhough, 2012, p. 6). Applying this to AVH, it can be hypothesized that if the original verbal memory of the trauma is reactivated, then it may be reconsolidated in altered form based on the current cognitive and affective state of the person. In this way, the contemporary emotions the voice-hearer has in relation to the trauma, which often involve guilt and shame (McCarthy-Jones, 2012; Corstens and Longden, 2013), could actively shape the representation in memory of the trauma, creating memories thematically related to the trauma, in turn seeding AVH thematically related to the trauma.

### Goals

One factor that may influence whether such memories come to consciousness (a pre-requisite for them being experienced as AVH) is the voice-hearer’s current goals. Conway and Pleydell-Pearce (2000) argue that patterns of activation continually arise and dissipate in the autobiographical knowledge base, and emerge as conscious memories when they can be linked to the person’s current goals. Goals may thus influence the frequency of AVH, as well as their content. The idea that the content of voices may be related to the voice-hearer’s goals was tested in a recent study by Varese et al. (2015). This was performed with 22 clinical voice-hearers and 18 non-clinical voice-hearers (clinical/non-clinical status being assessed using the facets of the Structural Clinical Interview for DSM-IV-TR; Axis I Disorder; First et al., 2002). Participants’ personal goals were assessed using a modified version of the Goal Task of Dickson and MacLeod (2004). Thematic associations between personal goals and voice-content were assessed using a variable-oriented coding procedure which focused on predefined, theory-driven variables and their relationships (Miles et al., 2014). A subsample of 20 coding matrices performed by the first author were also analyzed by a second coder. Inter-coder agreement was 95%, with kappa being 0.78, indicating a substantial agreement between coders. The study found that there was a thematic match between the content of voices and at least one reported personal goal in 83% of the total sample. Seventy percent of participants described voices that could be matched with two or more of their personal goals. For example, one participant’s personal goal was “Being a confident and competent person.” They heard a voice which said things such as “People think you are stupid,” “You don’t even know what you are talking about,” “You will never be good at anything.” Another participant had the goal of “Protect myself and my family,” and heard voices saying “You are going to have to watch yourself mate, because he is going to attack you. So why don’t you retaliate by grabbing hold of his neck and pushing him to the floor?” Goals (e.g., being safe) which are incongruent with earlier traumatic experiences, or whose development was spurred by earlier traumas, could hence be a source of AVH-content (whether this be through encouraging memories related to the trauma, or influencing ongoing inner speech or auditory verbal imagery; Jones and Fernyhough, 2007). The second example above, in which being safe was an important personal goal and relate to AVH content, can also be seen to link to another prominent theory of AVH, which is based in hypervigilance.

### Beyond Memory: Hypervigilance

Hypervigilance for threat, a characteristic sequel of trauma and symptom of PTSD (Dalgleish et al., 2001; American Psychiatric Association, 2013) has been proposed be a causative mechanism underpinning at least some AVH (Dodgson and Gordon, 2009; Garwood et al., 2015). This offers a route through which current concerns may generate content that is related to previous traumatic events, but which is not a direct memory of things heard. To illustrate this, consider the example of Rick, who was referred to an Early Intervention in Psychosis service (Smailes et al., submitted). Rick had been involved in a violent confrontation with a local gang, in which he was trying to protect his father. After this he became hypervigilant for any signs that he was to be targeted in a reprisal attack. As a result, he began to hear comments from people passing his house at night suggesting that he would be assaulted. His voices were clearly thematically related to his traumatic encounter with the gang, but were not direct re-experiences of things said during this. Instead, hypervigilance resulted in auditory false positives (Dodgson and Gordon, 2009) for words and phrases of concern to Rick, which were naturally related to the trauma, but not identical to it.

## Therapeutic Implications

Fernyhough (2012) has eloquently described how, “We remember the past through the lens of the present: what we believe now, what we want now. Memories might be about the past, but they are constructed in the present to suit the needs of the self.” If traumatic memories form the basis of some AVH, this suggests that changing people’s current beliefs and perspectives on themselves in relation to the trauma may both alter their memories they construct and the ability of voices to arise from these. The potential for trauma-based interventions to alter the negative content of voices is particularly important as there is currently no evidence that the dominant psychotherapy for voice-hearing, CBT for psychosis, is able to reduce the perceived malevolence of voices (Thomas et al., 2014; McCarthy-Jones et al., 2015). Could changes to how a person thinks and feels about an earlier traumatic event related to the voice alter the affective tone of the voice, e.g., changing malign voices into supportive ones?

Evidence for this is limited, but studies of compassion focussed therapy (CFT) are suggestive. CFT is based on theories of emotional regulation (Mayhew and Gilbert, 2008), and posits that hypersensitive threat processing (including threats to social-rank linked with shame and stigmatization and/or that originating from trauma exposure) can severely impair emotional regulation and reduce affiliation abilities. It therefore aims to help people develop the capacity for compassionate responding to self and others to help regulate emotion and mitigate distressing threat appraisals. In a small-scale case series, Mayhew and Gilbert (2008, p. 133) found CFT “had a major effect on…hostile voices, changing them into more reassuring, less persecutory and less malevolent voices.” Preliminary results of CFT for psychosis more generally are also encouraging (Braehler et al., 2013).

In addition to working with beliefs about the emotional event at the root of the voice(s), voices themselves may be directly engaged with, dialogically, in order to reconsolidate them into a more benevolent form. The exploratory technique of Voice Dialoguing (Corstens et al., 2011, 2012) is premised on a dissociative model that frames SZ-AVH as disowned experiential/emotional representations that can be directly engaged with by a therapist in a manner that instigates integration and reconciliation. The trauma literature likewise describes instances of dynamic engagement with voices (Holmes and Tinnin, 1995; Brewin, 2003). However, it should be noted that the efficacy of these approaches have not yet been demonstrated in a systematic, standardized manner with randomized samples of either PTSD or psychosis patients. The potential to eliminate SZ-AVH, if this is the hearer’s goal (Smailes et al., submitted), through addressing the traumatic life events underpinning them is also limited at the present time to tantalizing anecdotal evidence. For example, Leff et al. (2014) report that, in a trial of effectiveness of Avatar therapy for AVH, a participant’s voices ceased after guilt he felt over childhood sexual abuse he had experienced was removed by the therapist helping him to realize that what had happened was not his fault.

Whilst such suggestions are not new to PTSD interventions, they are novel for SZ-AVH, which are not traditionally viewed having content dynamically linked to earlier emotional traumas, but only being statically triggered by them. This suggests that CBT for psychosis, which currently focusses on changing beliefs about voices in order to reduce distress, may also benefit from the greater use of techniques that can change beliefs about any emotional events that inform AVH content (McCarthy-Jones, 2015). An essential first step would be to assess if the AVH is associated with an underpinning emotional trauma. In CBT terminology this would be accomplished with a longitudinal formulation (Johnstone and Dallos, 2006) or, using a more recent approach, creating a ‘construct’ of the voice that situates it in the context of a specific life-event (Romme and Escher, 2000; Corstens and Longden, 2013), thus encouraging association (rather than dissociation) between voice presence and adversity exposure, and providing a coherent framework for integrating dissimilated material into existing representational structures. In this respect, ‘deconstructing’ voices as latently psychologically interpretable/interpersonally significant is consistent with the premise that construing meaning and narrative from distressing experiences (including those in the context of psychosis) promotes hope, empowerment, reflectivity, and psychological adjustment (Romme and Escher, 2000; British Psychological Society [BPS], 2011). The next step would be to alter dysfunctional beliefs, not about the voices, but about the person’s role in any traumatic or emotional precursor events. This is already standard practice in the PTSD literature, where attributions of guilt and shame will often be worked with therapeutically. Techniques already used in PTSD such as imaginal rescripting and the integration of such experiences into autobiographical memory, may be beneficial to people with SZ-AVH. The development of psychological formulations would also allow the exploration of personal goals potentially underling the content of voices, and guide the provision of support to help clients achieve unmet goals that may be feeding voice content and/or occurrence (Varese et al., 2015).

A further technique which could potentially be imported from the trauma field for SZ-AVH is Eye Movement Desensitization and Reprocessing (EMDR). This is a robustly evidence-based therapy for PTSD (National Institute for Clinical Excellence [NICE], 2005; Foa et al., 2009), with provisional indications of utility in psychosis (Helen, 2011; van den Berg and van der Gaag, 2012; Van Der Vleugel et al., 2012), although there remains the need for controlled evaluations.

Another option may be to pharmacologically ‘defuse’ traumatic memories underpinning SZ-AVH (although this intervention raises a number of ethical concerns; President’s Council on Bioethics, 2003; Henry et al., 2007). Menzies (2012) found that five of six patients with PTSD, with memories ranging in age from four months to 31 years, all reported improvement when given propranolol shortly after having retrieved the traumatic memory. This included ‘fragmentation’ of the memory and difficulty accessing it, minimal or absent distress when thinking about it, and a feeling of emotional detachment, as if it were a normal non-traumatic memory or had happened to someone else. To our knowledge this has not been trialed with SZ-AVH to examine the effect of this on AVH.

The traditional role of antipsychotics in the treatment of SZ-AVH (Sommer et al., 2012) raises the potential risk that highlighting the equivalence of PTSD-AVH with many SZ-AVH could primarily be seen as suggesting the need to use antipsychotics for AVH and/or re-experiencing symptoms in PSTD, which is indeed now becoming increasingly common (Han et al., 2014). However, the insight that many SZ-AVH may have a similar cause to PTSD-AVH, namely trauma, argues more clearly for the importation of PTSD treatments into schizophrenia, rather than schizophrenia treatments into PTSD. Indeed, treatment-resistant schizophrenia is associated with a higher rate of lifetime adversities, with patients with four or more adversity exposures being four times more likely to find antipsychotics ineffective (Hassan and De Luca, 2015). This issue is further complicated by the potential for debilitating side-effects after long-term neuroleptic use (Ananth et al., 2004), and the elevated risk of suicide amongst patients experiencing distressing, treatment-refractory voices (Fialko et al., 2006).

Although we have focussed on PTSD and schizophrenia, clinical and conceptual overlaps between PTSD, psychosis, and dissociation have been explored by several theorists (Moskowitz and Corstens, 2007; Moskowitz et al., 2009; Craparo et al., 2014) with a number of hybrid diagnostic categories being proposed (e.g., dissociative subtypes of schizophrenia: Ross, 2008; and psychotic subtypes of PTSD: Braakman et al., 2009). Within such dissociative frameworks, AVH have been conceived as ‘disowned,’ disaggregated representations of past events (i.e., trauma-fixated) that aurally encroach on functioning-focused parts of the personality, consequently perceived as depersonalized and decontextualized stimuli (Moskowitz et al., 2009) that intrude into the executive self, wherein “disturbed connectivity or coherence…may negatively affect the normal patterns of synchronous activity…[that constitute] integrative functions of consciousness” (Bob and Mashour, 2011, p. 1046). Dissociation has been found to mediate the relation between childhood trauma and hallucinations in patients with schizophrenia-spectrum disorders (Varese et al., 2012a), demonstrating the utility of considering this concept in schizophrenia as well as PTSD. As psychosis patients are frequently excluded from treatment trials in PTSD and dissociative disorders, there remains limited data on the utility of applying dissociation-informed treatment protocols to individuals with complex comorbidity. As such, it should be noted that much existing evidence is therefore limited to small case series and observational data. An exception to this is non-randomized trials of PTSD-directed treatment in psychosis patients, which show that CBT programs based on cognitive restructuring (Rosenberg et al., 2004) and exposure paradigms (Frueh et al., 2009) provide clinical benefit for reducing posttraumatic symptoms without adverse effects.

Despite guidelines emphasizing the need to routinely assess adversity exposure in psychiatric service-users (National Health Service Confederation [NHS], 2008), such recommendations are not always adequately implemented (Hepworth and McGowan, 2013). While a history of maltreatment should not be presupposed, the evidence outlined here supports the contention that clinicians should receive adequate support and training for making routine enquiries about abuse and adversity. This is particularly important given the significant under-detection of posttraumatic stress in people with diagnoses of schizophrenia-spectrum disorders (Lommen and Restifo, 2009), and that such individuals are less likely to receive an appropriate clinical response (e.g., trauma-focussed interventions) relative to those with non-psychotic diagnoses (Agar and Read, 2002) especially in cases where clinicians hold strong causal beliefs about biogenetic etiology (Young et al., 2001).

## Conclusion

In this paper we firstly argued that AVH are regularly reported in both PTSD and schizophrenia, and furthermore that AVH are qualitatively similar in their phenomenology across these two diagnoses. One implication of this is the need to consider naming AVH as a characteristic symptom of PTSD, in addition to existing criterion of “Recurrent, involuntary, and intrusive memories” and “Dissociative reactions (e.g., flashbacks),” which share much in common with AVH and yet are distinct experiences. This is consistent with Bosson et al.’s (2011) contention that it is problematic that diagnostic guidelines for PTSD do not include criterion involving AVH that are related to but do not replicate the traumatic event.

Our second argument was that AVH in both PTSD and schizophrenia are often associated with earlier traumatic life-experiences (by definition in PTSD, but also commonly in people diagnosed schizophrenia). It is becoming clear that labeling AVH as a “psychotic symptom” is a Schneiderian hangover that the mind sciences need to sober up from. If AVH is viewed as a psychotic symptom, caused by schizophrenia-spectrum disorders, then it is naturally going to cause puzzlement when it is reported in people with non-psychotic diagnoses such as PTSD or borderline personality disorder. However, if AVH is viewed as often (although not always) being a post-traumatic reaction, then its presence in people with PTSD or borderline personality disorder (in which childhood trauma is common, and where AVH are also phenomenology similar to SZ-AVH; Kingdon et al., 2010) is to be expected. Rather than primarily searching for psychotic subtypes of PTSD or borderline personality disorder, future research may more profitably explore, (a) why trauma leads to AVH in some people but not others); (b) whether there exist trauma-based and non-trauma based subtypes of AVH in schizophrenia, each with a characteristic biological phenotype (such as an inflammatory phenotype in the case of the former; Dennison et al., 2012); and (c) why some AVH are more likely to be complicated by delusional elaboration than others (and are hence more likely to result in a diagnosis of schizophrenia), a process already tentatively linked with environmental risk factors and affective dysregulation (Smeets et al., 2012).

Our third argument was that the content of AVH in both PTSD and schizophrenia are related to trauma (when present in schizophrenia) in a similar way, most commonly in a manner involving indirect or directs links, and not involving re-experiencing. Starting from well-established cognitive models of re-experiencing symptoms in PTSD, we explored how contemporary models of memory could account for the nature of this relationship. We suggested this may be due to the memories of verbal material pertaining to trauma being shaped by the person’s current thoughts and emotions pertaining to the trauma. We also noted that the intrusion into consciousness of this memory could be facilitated by linkages with current personal goals, as well as noting that other forms of mentation (i.e., inner speech, auditory verbal imagery) could also potentially be triggered by personal goals and form the source of AVH. Finally, we highlighted that hypervigilance following trauma could be another way to account for why AVH often have thematic links to earlier emotionally overwhelming events, without being re-experiences of them.

Building on these arguments, we proposed that psychotherapies that aim to formulate and revise unhelpful beliefs about any emotional events that underpin AVH may be beneficial in improving the affective valence of the voice, reducing accompanying distress through breaking down dissociative barriers, and encouraging psychological unity and integration. This review identifies important rationales for extending knowledge from the trauma literature more vigorously into psychosis research. An important caveat is that not all SZ-AVH may be related to earlier traumas, even when such traumas are present (Hardy et al., 2005). Although it may be particularly tempting to link SZ-AVH that have negative emotional content to earlier experiences of trauma, not only are there other potential reasons available for why SZ-AVH may have negative content (e.g., Sommer and Diederen, 2009), but it should be noted that Hardy et al. (2005) found that their participants with a history of trauma were equally likely as participants with no history of trauma to have voices with content involving themes of humiliation, guilt, or threat (although as we noted above, this study did not examine links between AVH and emotionally overwhelming events that we not assessed by the trauma questionnaire employed by their study). Research into SZ-AVH therefore needs to be open to the concept of equifinality (Cicchetti and Rogosch, 1996) which, as applied here, allows that there may be multiple aetiological routes to phenomenologically similar AVH, of which trauma may be only one. The developmental routes which lead to AVH, and the interaction of biological and environment events along these pathways, is in need of much greater understanding. Despite these caveats, it is nonetheless clear that the clinical exploration of potential links between AVH-content and trauma (or emotionally overwhelming events in general), the implementation of (trauma-specialized) psychotherapeutic interventions where appropriate, and the dismantling of the iron curtain between AVH in schizophrenia and AVH in PTSD, should have beneficial consequences for many patients. Psychotic and dissociative voice-hearing will likely, in many cases, turn out to be the same thing.

## Conflict of Interest Statement

The authors declare that the research was conducted in the absence of any commercial or financial relationships that could be construed as a potential conflict of interest.

## References

[B1] Agar K. Read J. (2002). What happens when people disclose sexual or physical abuse to staff at a community mental health centre? *Int. J. Ment. Health Nurs*. 11 70–79. 10.1046/j.1440-0979.2002.00230.x 12430188

[B2] American Psychiatric Association. (2000). *Diagnostic and Statistical Manual of Mental Disorders* 4th Edn (Washington, DC: American Psychiatric Association).

[B3] American Psychiatric Association. (2013). *Diagnostic and Statistical Manual of Mental Disorders* 5th Edn (Washington, DC: American Psychiatric Association).

[B4] Ananth J. Parameswaran S. Gunatilake S. (2004). Side effects of atypical antipsychotic drugs. *Curr. Pharm. Des.* 10 2219–2229. 10.2174/1381612043384088 15281897

[B5] Anketell C. Dorahy M. J. Curran D. (2011). A preliminary qualitative investigation of voice hearing and its association with dissociation in chronic PTSD. *J. Trauma Dissociation* 12 88–101. 10.1080/15299732.2010.514844 21240740

[B6] Anketell C. Dorahy M. J. Shannon M. Elder R. Hamilton G. Corry M. (2010). An exploratory analysis of voice hearing in chronic PTSD: potential associated mechanisms. *J. Trauma. Dissociation* 11 93–107. 10.1080/15299730903143600 20063251

[B7] Bartlett F. C. (1932). *Remembering: A Study in Experimental and Social Psychology.* Cambridge: Cambridge University Press.

[B8] Bentall R. P. Wickham S. Shevlin M. Varese F. (2012). Do specific early-life adversities lead to specific symptoms of psychosis? A study from the 2007 the Adult Psychiatric Morbidity Survey. *Schizophr Bull.* 38 734–740. 10.1093/schbul/sbs049 22496540 PMC3406525

[B9] Bleich A. Moskowits L. (2000). Post traumatic stress disorder with psychotic features. *Croat. Med. J.* 41 442–445. 11063771

[B10] Bob P. Mashour G. A. (2011). Schizophrenia, dissociation, and consciousness. *Conscious. Cogn.* 20 1042–1049. 10.1016/j.concog.2011.04.013 21602061

[B11] Bosson J. V. Reuther E. T. Cohen A. S. (2011). The comorbidity of psychotic symptoms and posttraumatic stress disorder: evidence for a specifier in DSM-5. *Clin. Schizophr. Relat. Psychoses* 5 147–154. 10.3371/CSRP.5.3.5 21983499

[B12] Braakman M. H. Kortmann F. A. van den Brink W. (2009). Validity of ‘post-traumatic stress disorder with secondary psychotic features’: a review of the evidence. *Acta Psychiatr. Scand.* 119 15–24. 10.1111/j.1600-0447.2008.01252.x 18764840

[B13] Braehler C. Gumley A. Harper J. Wallace S. Norrie J. Gilbert P. (2013). Exploring change processes in compassion focused therapy in psychosis: results of a feasibility randomized controlled trial. *Br. J. Clin. Psychol.* 52 199–214. 10.1111/bjc.12009 24215148

[B14] Brewin C. (2001). A cognitive neuroscience account of posttraumatic stress disorder and its treatment. *Behav. Res. Ther.* 39 373–393. 10.1016/j.jpsychires.2012.05.006 11280338

[B15] Brewin C. R. (2003). *Posttraumatic Stress Disorder: Malady or Myth*? New Haven, CT: Yale University Press.

[B16] Brewin C. R. Dalgleish T. Joseph S. (1996). A dual representation theory of post traumatic stress disorder. *Psychol. Rev.* 103 670–686. 10.1016/j.jbtep.2013.07.011 8888651

[B17] Brewin C. R. Patel T. (2010). Auditory pseudohallucinations in United Kingdom war veterans and civilians with posttraumatic stress disorder. *J. Clin. Psychiatry* 71 419–425. 10.4088/JCP.09m05469blu 20361915

[B18] British Psychological Society [BPS]. (2011). *Good Practice Guidelines on the use of Psychological Formulation. A Report by the British Psychological Society Division of Clinical Psychology.* Leicester: British Psychological Society.

[B19] Butler R. W. Mueser K. T. Sprock J. Braff D. L. (1996). Positive symptoms of psychosis in posttraumatic stress disorder. *Biol. Psychiatry* 39 839–844. 10.1016/0006-3223(95)00314-2 9172704

[B20] Cicchetti D. Rogosch F. A. (1996). Equifinality and multifinality in developmental psychopathology. *Dev. Psychopathol.* 8 597–600. 10.1017/S0954579400007318

[B21] Conway M. A. Pleydell-Pearce C. W. (2000). The construction of autobiographical memories in the self-memory system. *Psychol. Rev.* 107 261–288. 10.1037/0033-295X.107.2.261 10789197

[B22] Corstens D. Longden E. (2013). The origins of voices: links between voice hearing and life history in a survey of 100 cases. *Psychosis* 5 270–285. 10.1080/17522439.2013.816337

[B23] Corstens D. Longden E. May R. (2012). Talking with voices: exploring what is expressed by the voices people hear. *Psychosis* 4 95–104. 10.1080/17522439.2011.571705

[B24] Corstens D. May R. Longden E. (2011). “Talking with voices,” in *Psychosis as a Personal Crisis: An Experience Based Approach* eds Romme M. Escher S. (London: Routledge) 166–178.

[B25] Craparo G. Gori A. Mazzola E. Petruccelli I. Pellerone M. Rotondo G. (2014). Posttraumatic stress symptoms, dissociation, and alexithymia in an Italian sample of flood victims. *Neuropsychiatr. Dis. Treat.* 10 2281–2284. 10.2147/NDT.S74317 25489247 PMC4257106

[B26] Dalgleish T. Moradi A. R. Taghavi M. R. Neshat-Doost H. T. Yule W. (2001). An experimental investigation of hypervigilance for threat in children and adolescents with post-traumatic stress disorder. *Psychol Med.* 31 541–547. 10.1017/S0033291701003567 11305862

[B27] Dennison U. McKernan D. Cryan J. Dinan T. (2012). Schizophrenia patients with a history of childhood trauma have a pro-inflammatory phenotype. *Psychol. Med.* 42 1865–1871. 10.1017/S0033291712000074 22357348

[B28] Dickson J. M. MacLeod A. K. (2004). Approach and avoidance goals and plans: their relationship to anxiety and depression. *Cognit. Ther. Res.* 28 415–432. 10.1023/B:COTR.0000031809.20488.ee

[B29] Dodgson G. Gordon S. (2009). Avoiding false negatives: are some auditory hallucinations an evolved design flaw? *Behav. Cogn. Psychother*. 37 325–334. 10.1017/S1352465809005244 19371459

[B30] Ehlers A. Clark D. M. (2000). A cognitive model of posttraumatic stress disorder. *Behav. Res. Ther.* 38 319–345. 10.1016/s0005-7967(99)00123-0 10761279

[B31] Fernyhough C. (2012). *Pieces of Light: The New Science of Memory* Kindle Edition. London: Profile Books.

[B32] Fialko L. Freeman D. Bebbington P. E. Kuipers E. Garety P. A. Dunn G. (2006). Understanding suicidal ideation in psychosis: findings from the psychological prevention of relapse in psychosis (PRP) trial. *Acta Psychiatr. Scand.* 114 177–186. 10.1111/j.1600-0447.2006.00849.x 16889588

[B33] First M. B. Spitzer R. L. Gibbon M. Williams J. B. W. (2002). *Structured Clinical Interview for DSM-IV-TR Axis I Disorders, Research Version* Patient Edition New York, NY: Biometrics Research.

[B34] Foa E. B. Keane T. M. Friedman M. J. (2009). *Effective Treatments for PTST: Practice Guidelines of the International Society for Traumatic Stress Studies.* New York, NY: Guilford Press.

[B35] Fowler D. Freeman D. Steel C. Hardy A. Smith B. Hackman C. (2006). “The catastrophic interaction hypothesis: how do stress, trauma, emotion and information processing abnormalities lead to psychosis?,” in *Trauma and Psychosis: New Directions for Theory and Therapy Larkin* ed. Morrison W. (London: Routledge) 101–124.

[B36] Frueh B. C. Grubaugh A. L. Cusack K. J. Kimble M. O. Elhai J. D. Knapp R. G. (2009). Exposure-based cognitive-behavioral treatment of PTSD in adults with schizophrenia or schizoaffective disorder: a pilot study. *J. Anxiety Disord.* 23 665–675. 10.1016/j.janxdis.2009.02.005 19342194 PMC2737503

[B37] Garwood L. Dodgson G. Bruce V. McCarthy-Jones S. (2015). A preliminary investigation into the existence of a hypervigilance subtype of auditory hallucination in people with psychosis. *Behav. Cogn. Psychother.* 43 52–62. 10.1017/S1352465813000714 23962410

[B38] Green B. L. (1996). “Trauma History Questionnaire,” in *Measurement of Stress, Self-Report Trauma and Adaptation* ed. Stamm B. H. (Lutervile, MD: Sidran Press) 366–368.

[B39] Hamner M. B. Frueh B. C. Ulmer H. G. Huber M. G. Twomey T. J. Tyson C. (2000). Psychotic features in chronic posttraumatic stress disorder and schizophrenia: comparative severity. *J. Nerv. Ment. Dis.* 188 217–221. 10.1097/00005053-200004000-00004 10789998

[B40] Han C. Pae C.-U. Wang S.-M. Lee S.-J. Patkar A. A. Masand P. S. (2014). The potential role of atypical antipsychotics for the treatment of posttraumatic stress disorder. *J. Psychiat. Res.* 56 72–81. 10.1016/j.jpsychires.2014.05.003 24882700

[B41] Hardy A. Fowler D. Freeman D. Smith B. Steel C. Evans J. (2005). Trauma and hallucinatory experiences in psychosis. *J. Nerv. Ment. Dis.* 193 501–507. 10.1097/01.nmd.0000172480.56308.21 16082293

[B42] Hassan A. N. De Luca V. (2015). The effect of lifetime adversities on resistance to antipsychotic treatment in schizophrenia patients. *Schizophr. Res.* 161 496–500. 10.1016/j.schres.2014.10.048 25468176

[B43] Helen. (2011). Child abuse and voice hearing: finding healing through EMDR. *Psychosis* 3 90–95. 10.1080/17522439.2010.542827

[B44] Henry M. Fishman J. R. Youngner S. J. (2007). Propranolol and the prevention of post-traumatic stress disorder: is it wrong to erase the “sting” of bad memories? *Am. J. Bioethics* 7 12–20. 10.1080/15265160701518474 17849331

[B45] Hepworth L. McGowan L. (2013). Do mental health professionals enquire about childhood sexual abuse during routine mental health assessment in acute mental health settings? A substantive literature review. *J. Psychiatr. Ment. Health Nurs.* 20 473–483. 10.1111/j.1365-2850.2012.01939.x 22702227

[B46] Holmes D. S. Tinnin L. W. (1995). The problem of auditory hallucinations in combat PTSD. *Traumatology* 1 1 10.1177/153476569500100201

[B47] Hupbach A. Gomez R. Hardt O. Nadel L. (2007). Reconsolidation of episodic memories: a subtle reminder triggers integration of new information. *Learn. Mem.* 14 47–53. 10.1101/lm.365707 17202429 PMC1838545

[B48] Jessop M. Scott J. Nurcombe B. (2008). Hallucinations in adolescent inpatients with post-traumatic stress disorder and schizophrenia: similarities and differences. *Australas Psychiatry* 16 268–272. 10.1080/10398560801982580 18608156

[B49] Johnstone L. Dallos R. (2006). *Formulation in Psychology and Psychotherapy: Making Sense of People’s Problems.* London: Routledge.

[B50] Jones S. R. Fernyhough C. (2007). Neural correlates of inner speech and auditory verbal hallucinations: a critical review and theoretical integration. *Clin. Psychol. Rev.* 27 140–154. 10.1016/j.cpr.2006.10.001 17123676

[B51] Kent G. Wahass S. (1996). The content and characteristics of auditory hallucinations in Saudi Arabia and the UK: a cross-cultural comparison. *Acta Psychiatr. Scand.* 94 433–437. 10.1111/j.1600-0447.1996.tb09886.x 9020995

[B52] Kingdon D. G. Ashcroft K. Bhandari B. Gleeson S. Warikoo N. Symons M. (2010). Schizophrenia and borderline personality disorder: similarities and differences in the experience of auditory hallucinations, paranoia, and childhood trauma. *J. Nerv. Ment. Dis.* 198 399–403. 10.1097/NMD.0b013e3181e08c27 20531117

[B53] Leff J. Williams G. Huckvale M. Arbuthnot M. Leff A. P. (2014). Avatar therapy for persecutory auditory hallucinations: what is it and how does it work? *Psychosis* 6 166–176. 10.1080/17522439.2013.77345 24999369 PMC4066885

[B54] Loftus E. F. (2005). Planting misinformation in the human mind: a 30-year investigation of the malleability of memory. *Learn. Mem.* 12 361–366. 10.1101/lm.94705 16027179

[B55] Lommen M. J. J. Restifo K. (2009). Trauma and posttraumatic stress disorder (PTSD) in patients with schizophrenia or schizoaffective disorder. *Commun. Ment. Health J.* 45 485–496. 10.1007/s10597-009-9248-x PMC2791484 19777347

[B56] Mayhew S. L. Gilbert P. (2008). Compassionate mind training with people who hear malevolent voices: a case series report. *Clin. Psychol. Psychother.* 15 113–138. 10.1002/cpp.566 19115433

[B57] McCarthy-Jones S. (2011). Voices from the storm: a critical review of quantitative studies of auditory verbal hallucinations and childhood sexual abuse. *Clin. Psychol. Rev.* 31 983–992. 10.1016/j.cpr.2011.05.004 21736865

[B58] McCarthy-Jones S. (2012). *Hearing Voices: The Histories, Meanings and Causes of Auditory Verbal Hallucinations.* Cambridge, MA: Cambridge University Press 10.1017/CBO9781139017534

[B59] McCarthy-Jones S. (2015). “What have we learnt about the phenomenology of voice-hearing,” in *Hallucinations: From Theory to Therapy* eds Hayward M. Strauss C. McCarthy-Jones S. (London: Taylor & Francis) 5–26.

[B60] McCarthy-Jones S. Thomas N. Dodgson G. Fernyhough C. Brotherhood E. Wilson G. (2015). “What have we learnt about the ability of cognitive behavioural therapy to help with voice-hearing?,” in *Hallucinations: From Theory to Therapy* eds Hayward M. Strauss C. McCarthy-Jones S. (London: Taylor and Francis).

[B61] McCarthy-Jones S. Trauer T. Mackinnon A. Sims E. Thomas N. Copolov D. L. (2014). A new phenomenological survey of auditory hallucinations: evidence for subtypes and implications for theory and practice. *Schizophr. Bull.* 40 231–235. 10.1093/schbul/sbs156 23267192 PMC3885292

[B62] McGaugh J. L. (2000). Memory–a century of consolidation. *Science* 287 248–251. 10.1038/nn739 10634773

[B63] Menzies R. P. (2012). Propranolol, traumatic memories, and amnesia: a study of 36 cases. *J. Clin. Psychiatry* 73 129–130. 10.4088/JCP.11l07121 22316579

[B64] Miles M. B. Huberman A. M. Saldana J. (2014). *Qualitative Data Analysis: A Method Sourcebook.* London: Sage.

[B65] Moskowitz A. Corstens D. (2007). Auditory hallucinations: psychotic symptom or dissociative experience? *J. Psychol. Trauma*. 6 35–63. 10.1300/J513v06n02_04

[B66] Moskowitz A. Read J. Farrelly S. Rudegeair T. Williams O. (2009). “Are psychotic symptoms traumatic in origin and dissociative in kind?,” in *Dissociation and the Dissociative Disorders: DSM-V and Beyond* eds Dell P. F. O’Neill J. A. (New York, NY: Routledge) 521–533.

[B67] National Health Service Confederation [NHS]. (2008). *Briefing 162: Implementing National Policy on Violence and Abuse.* London: National Health Service Confederation.

[B68] National Institute for Clinical Excellence [NICE]. (2005). *Post Traumatic Stress Disorder (PTSD): The Management of Adults and Children in Primary and Secondary Care.* London: NICE Guidelines.

[B69] Nayani T. H. David A. S. (1996). The auditory hallucination: a phenomenological survey. *Psychol. Med.* 26 177–189. 10.1017/S003329170003381X 8643757

[B70] President’s Council on Bioethics. (2003). *Beyond Therapy: Biotechnology and the Pursuit of Happiness.* Washington, DC: President’s Council on Bioethics, Dana Press.

[B71] Raune D. Bebbington P. Dunn G. Kuipers E. (2006). Event attributes and the content of psychotic experiences in first-episode psychosis. *Psychol. Med.* 36 221–230. 10.1017/S003329170500615X 16336724

[B72] Read J. Agar K. Argyle N. Aderhold V. (2003). Sexual and physical abuse during childhood and adulthood as predictors of hallucinations, delusions and thought disorder. *Psychol. Psychother.* 76 1–22. 10.1348/14760830260569210.2003 12689431

[B73] Read J. Fosse R. Moskowitz A. Perry B. (2014). The traumagenic neurodevelopmental model of psychosis revisited. *Neuropsychiatry* 4 65–79. 10.2217/npy.13.89

[B74] Read J. van Os J. Morrison A. P. Ross C. A. (2005). Childhood trauma, psychosis and schizophrenia: a literature review with theoretical and clinical implications. *Acta Psychiatr. Scand.* 112 330–350. 10.1111/j.1600-0447.2005.00634.x 16223421

[B75] Reiff M. Castille D. M. Muenzenmaier K. Link B. (2012). Childhood abuse and the content of adult psychotic symptoms. *Psychol. Trauma* 4 356–369. 10.1037/a0024203

[B76] Romme M. Escher S. (2000). *Making sense of Voices.* London: Mind Publications.

[B77] Rosenberg S. D. Mueser K. T. Jankowski K. Salyers M. P. Acker K. (2004). Cognitive-behavioral treatment of PTSD in severe mental illness: results of a pilot study. *Am. J. Psychiatr. Rehabil.* 7 171–186. 10.1080/15487760802615863

[B78] Ross C. A. (2008). “Dissociative schizophrenia,” in *Psychosis, Trauma and Dissociation: Emerging Perspectives on Severe Psychopathology* eds Moskowitz A. Schäfer I. Dorahy M. J. (Chichester: John Wiley & Sons, Ltd) 281–294. 10.1002/9780470699652.ch20

[B79] Schneider K. (1959). *Clinical Psychopathology.* New York: Grune and Stratton.

[B80] Scott J. G. Nurcombe B. Sheridan J. McFarland M. (2007). Hallucinations in adolescents with post-traumatic stress disorder and psychotic disorder. *Australas Psychiatry* 15 44–48. 10.1080/10398560601083084 17464634

[B81] Sheffield J. M. Williams L. E. Blackford J. U. Heckers S. (2013). Childhood sexual abuse increases risk of auditory hallucinations in psychotic disorders. *Compr. Psychiatry* 54 1098–1104. 10.1016/j.comppsych.2013.05.013 23815887 PMC3779472

[B82] Smeets F. Lataster T. Hommes J. Lieb R. Wittchen H. U. van Os J. (2012). Evidence that onset of psychosis in the population reflects early hallucinatory experiences that through environmental risks and affective dysregulation become complicated by delusions. *Schizophr. Bull.* 38 531–542. 10.1093/schbul/sbq117 21030456 PMC3329993

[B83] Smith B. Fowler D. G. Freeman D. Bebbington P. Bashforth H. Garety P. (2006). Emotion and psychosis: links between depression, self-esteem, negative schematic beliefs and delusions and hallucinations. *Schizophr. Res.* 86 181–188. 10.1016/j.schres.2006.06.018 16857346

[B84] Sommer I. Diederen K. (2009). Language production in the non-dominant hemisphere as a potential source of auditory verbal hallucinations. *Brain* 132 1–2. 10.1093/brain/awp04019168453

[B85] Sommer I. E. Slotema C. W. Daskalakis Z. J. Derks E. M. Blom J. D. van der Gaag M. (2012). The treatment of hallucinations in schizophrenia spectrum disorders. *Schizophr. Bull.* 38 704–714. 10.1093/schbul/sbs034 22368234 PMC3577047

[B86] Thomas N. Hayward M. Peters E. van der Gaag M. Bentall R. P. Jenner J. (2014). Psychological therapies for auditory verbal hallucinations (voices): current status and key research directions. *Schizophr. Bull.* 40 S202–S212. 10.1093/schbul/sbu037 24936081 PMC4141318

[B87] Thomas P. Mathur P. Gottesman I. I. Nagpal R. Nimgaonkar V. L. Deshpande S. N. (2007). Correlates of hallucinations in schizophrenia: a cross-cultural evaluation. *Schizophr. Res.* 92 41–49. 10.1016/j.schres.2007.01.017 17350224 PMC5443754

[B88] Thompson A. Nelson B. McNab C. Simmons M. Leicester S. McGorry P. D. (2010). Psychotic symptoms with sexual content in the “ultra high risk” for psychosis population: frequency and association with sexual trauma. *Psychiatr. Res.* 177 84–91. 10.1016/j.psychres.2010.02.011 20304504

[B89] van den Berg D. P. van der Gaag M. (2012). Treating trauma in psychosis with EMDR: a pilot study. *J. Behav. Ther. Exp. Psychiatry* 43 664–671. 10.1016/j.jbtep.2011.09.011 21963888

[B90] Van Der Vleugel B. M. van den Berg D. P. G. Staring A. B. P. (2012). Trauma, psychosis, post-traumatic stress disorder and the application of EMDR. *Riv. Psichiatr.* 47(Suppl. 2), 33–38. 10.1708/1071.11737 22622277

[B91] Varese F. Barkus E. Bentall R. P. (2012a). Dissociation mediates the relationship between childhood trauma and hallucination-proneness. *Psychol Med.* 42 1025–1036. 10.1017/S0033291711001826 21896238

[B92] Varese F. Smeets F. Drukker M. Lieverse R. Lataster T. Viechtbauer W. (2012b). Childhood trauma increases the risk of psychosis: a meta-analysis of patient-control, prospective- and cross sectional cohort studies. *Schizophr. Bull.* 38 661–671. 10.1016/j.jpsychires.2012.01.023 22461484 PMC3406538

[B93] Varese F. Tai S. J. Pearson L. Mansell W. (2015). Thematic associations between personal goals and clinical and non-clinical voices (auditory verbal hallucinations). *Psychosis* (in press). 10.1080/17522439.2015.1040442

[B94] Waters F. A. Badcock J. C. Maybery M. T. (2006). The ’who’ and ’when’ of context memory: different patterns of association with auditory hallucinations. *Schizophr. Res.* 82 271–273. 10.1016/j.schres.2005.12.847 16417987

[B95] Wichert S. Wolf O. T. Schwabe L. (2013). Updating of episodic memories depends on the strength of new learning after memory reactivation. *Behav. Neurosci.* 127 331–338. 10.1037/a0032028 23458404

[B96] Young M. Read J. Barker-Collo S. Harrison R. (2001). Evaluating and overcoming barriers to taking abuse histories. *Prof. Psychol. Res. Pract.* 32 407–414. 10.1037//0735-7028.32.4.407

